# Traumatology: Adoption of the Sm@rtEven Application for the Remote Evaluation of Patients and Possible Medico-Legal Implications

**DOI:** 10.3390/jcm11133644

**Published:** 2022-06-23

**Authors:** Giuseppe Basile, Riccardo Accetta, Susanna Marinelli, Riccardo D’Ambrosi, Quirino Alessandro Petrucci, Arianna Giorgetti, Alessandro Nuara, Simona Zaami, Stefania Fozzato

**Affiliations:** 1 IRCCS Galeazzi Orthopedics Institute, 20161 Milano, Italy; basiletraumaforense@gmail.com (G.B.); riccacc@gmail.com (R.A.); riccardo.dambrosi@hotmail.it (R.D.); alenuara91@gmail.com (A.N.); ste.fozzato@gmail.com (S.F.); 2 School of Law, Università Politecnica delle Marche, 60121 Ancona, Italy; susanna.marinelli@tiscali.it; 3 Department of Anatomical, Histological, Forensic and Orthopedic Sciences, “Sapienza” University of Rome, 00161 Rome, Italy; quirinoalessandro.petrucci@uniroma1.it; 4 Department of Medical and Surgical Sciences, Section of Legal Medicine, University of Bologna, 40126 Bologna, Italy; ari.giorgetti@gmail.com

**Keywords:** telemedicine, health services, remote follow-up, lower limb fracture, rehabilitation, European Union regulatory framework, forensic medicine

## Abstract

Telemedicine is the combination of technologies and activities that offer new remote ways of medical care. The Sm@rtEven application project is a remote assistance service that follows patients affected by lower limb fractures surgically treated at Galeazzi Orthopedic Institute (Milan, Italy). The Sm@rtEven application aims to evaluate the clinical conditions of patients treated for lower limb fracture after discharge from hospital using remote follow-up (FU). The project is not a substitute for traditional clinical consultations but an additional tool for a more complete and prolonged view over time. The Sm@rtEven application is installed on patients’ smartphones and is used daily to communicate with healthcare personnel. In the first protocol, patients had to complete different tasks for 30 days, such as monitoring the load progression on the affected limb, the number of steps during the day, and body temperature and completing a questionnaire. A simplified protocol was proposed due to the pandemic and logistical issues. The revised protocol enrolled patients after more than 30 days of their operation, prioritized the rehabilitation phase, and required patients to use the app for fewer days. After an initial phase of correct use, a reduction in patient compliance was gradually reported in the first protocol. However, patient compliance in the second protocol remained high (96.25%) in the recording of all the required parameters. The Sm@rtEven application has proven to be a valuable tool for following patients remotely, especially during the pandemic. Telemedicine has the same value as traditional clinical evaluations, and it enables patients to be followed over long distances and over time, minimizing any discomfort.

## 1. Introduction

Telemedicine is the combination of technologies and activities that offer new ways of providing medical care [1,2]. The technological development of the last few years has allowed a transformation of the healthcare sector. These advances could offer individualized, participatory, and preventive health services. In this context, mobile applications (apps) are a very promising element. Healthcare apps can be useful for patients and their caregivers or family members and also for healthcare professionals. In clinical practice, healthcare applications can have several functions: they can provide information on the pathology affecting the patient; allow doctors to evaluate the patients’ physical and functional state, mobility, motor skills, and emotional and psychological state; and help medical staff in the early recognition of the onset of new diseases, in the management of patients with motor disabilities, and in the implementation of pharmacological and rehabilitation programs [3,4,5,6,7,8,9].

The Sm@rtEven application project is a remote assistance service created by I-Tel, GOMEISA S.r.l., and the medical and managerial staff of Galeazzi Orthopedic Institute (Milan, Italy) to remotely evaluate and monitor patients affected by fractures of the lower limb who were surgically treated at the Traumatology and First Aid Department of the Galeazzi Orthopedic Institute. Remote monitoring was entrusted to medical and nursing staff who were qualified for the management of patients with acute and chronic wounds.

The aim of the Sm@rtEven project is to evaluate, over time and at home, the clinical conditions of patients treated surgically for a lower limb fracture after discharge from the hospital. The service, through the synergistic connection and interaction of different professional figures and through direct, telematic, and continuous communication between the patient and healthcare professionals, allows personalized care and the intensification of follow-up, in addition to guaranteeing patient care continuity. Once the patient is enrolled, an application is installed on their mobile device, to be used daily, through which they can communicate with healthcare personnel. The project should not be intended as a substitute for traditional clinical consultations, which are based on direct observation by doctors and nurses, but as an additional tool for a more complete and prolonged view over time.

## 2. Materials and Methods

**Figure 1:**
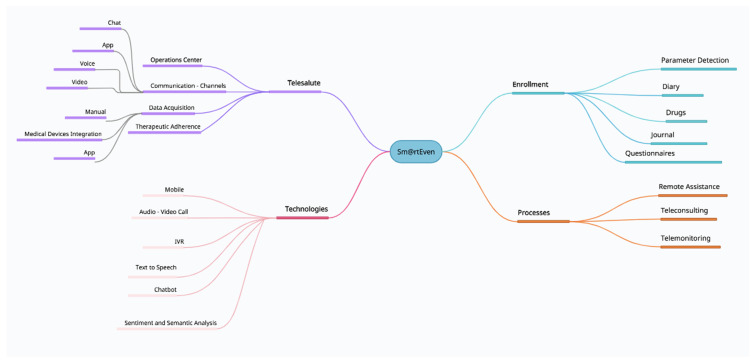
The different components involved in the functions of the Smart@Even application have been graphically represented. (IVR: interactive voice response).

This project is by no means intended to replace a first aid service and requires the active involvement of the patients who are to be monitored, the doctors who selected them for enrolment according to inclusion criteria, and the professional nurses who provide the information and clarifications necessary for nursing management through a digital platform. The doctors and nurses involved in the program answered the patients’ questions directly from 8 am to 8 pm and within 24 h for the rest of the day. The different components involved in the Smart@Even application functions have been graphically represented (Figure 1).

Enrolled patients, both males and females, had a lower limb fracture and met the following requirements:

− Has undergone reduction and synthesis surgery;
− 18–75 age range;
− Absence of degenerative and oncological cognitive conditions;
− Absence of language barriers;
− Has a smartphone with an Android operating system.

The exclusion criteria are a prosthetic replacement in the affected limb, a disability that precludes the use of the assigned devices, and functional motor limitation prior to the traumatic event.

The parameters analyzed in this project are:

− Progression of the load on the affected limb measured in kg (weight);
− Number of steps taken by the patient during the day;
− Body temperature;
− Administration of a daily evaluation questionnaire.

In order to evaluate the different parameters, the enrolled patients were asked to install the Sm@rtEven application on their smartphone during the observation period through a temporary https, active for the entire duration of the study (https://galeazzi.i-tel.it/appsmartsuitegate/app_even.jsp last accessed on 1 September 2021).

Patients were assigned two wireless devices, an electronic scale and an activity tracker, to evaluate some of the parameters considered. The scale was used to measure the progression of the load on the operated limb. In patients with musculoskeletal injuries of the lower limbs, a gradual and correct increase in axial load helps to maintain muscle mass and stimulates bone recovery and healing [10]. In clinical practice, bathroom scales have been used for weight-bearing purposes for many years. The patient is asked to step on the scale with the affected limb, remember the sensation of a certain weight, and try to reproduce it when moving on crutches. The sensation of the allowed weight is calculated as a percentage of the patient’s body weight and is progressively increased over time by the physician. The activity tracker is a bracelet to measure the number of steps taken by the patient during the day. The data measured by the two wireless devices were automatically recorded due to their synchronization with the patient’s smartphone through a Bluetooth connection. 

In addition, the patients were instructed to record their body temperature on the app, measured with an analogic thermometer, and to respond, through the app, to a multiple-choice questionnaire about their state of health. Once the requested data is entered, confirmation is requested from the patient, and the results are sent to an application control system (named Telesalute (i.e., telehealth)) to be viewed by the nurses involved. The Telesalute center is an internet platform that enables medical and nursing staff to view the patient’s anamnestic clinical data and the data entered daily by the patient and to program the set of parameters to be detected. 

The dashboard is a centralized tool from which the operator can check therapeutic progress and the parameters considered and handle any notifications and alarms, highlighted with different icons and color codes. It enables the supervision of the patient’s levels of adherence to the protocol by monitoring the recording times of the parameters. Furthermore, it allows for the overview and analysis of paths derived from parameter measurement and requests additional measurements whenever necessary.

**Figure 2:**
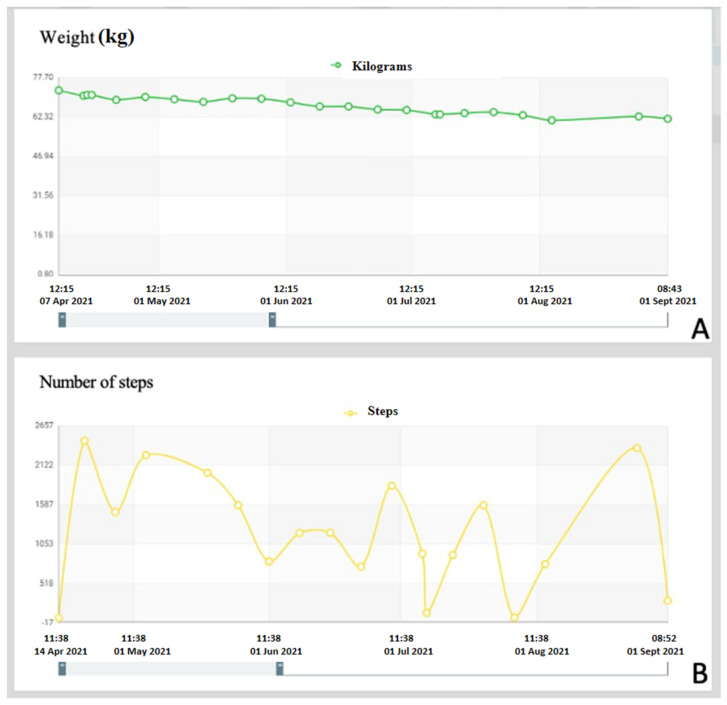
Examples of graphs obtained through the Telesalute dashboard. (**A**) Example of graphic representation relating to the progression of the load on the affected limb recorded by the patient. (**B**) Example of graphic representation relating to the number of steps recorded by the patient during the day.

On the Telesalute dashboard, graphs can be produced to show the trend of the variations of the individual parameters in order to gain a broad-ranging view of each individual patient (Figure 2A,B).

Patients receive daily notifications on their smartphones that indicate the activities to be carried out in order to facilitate the collection of the requested data. Another feature of the application is the ability to send notes, images, documents, and audio and multimedia files to the Telesalute control center in real-time.

### 2.1. Sm@rtEven Application System

Once the application is installed, the patient logs in by entering the credentials assigned by the recruiter doctor. The system stores the entered password directly to avoid possible forgetfulness. During first access, consent is required to view and utilize the multimedia contents of the device, geolocation, and the recording of audio and video.

**Figure 3:**
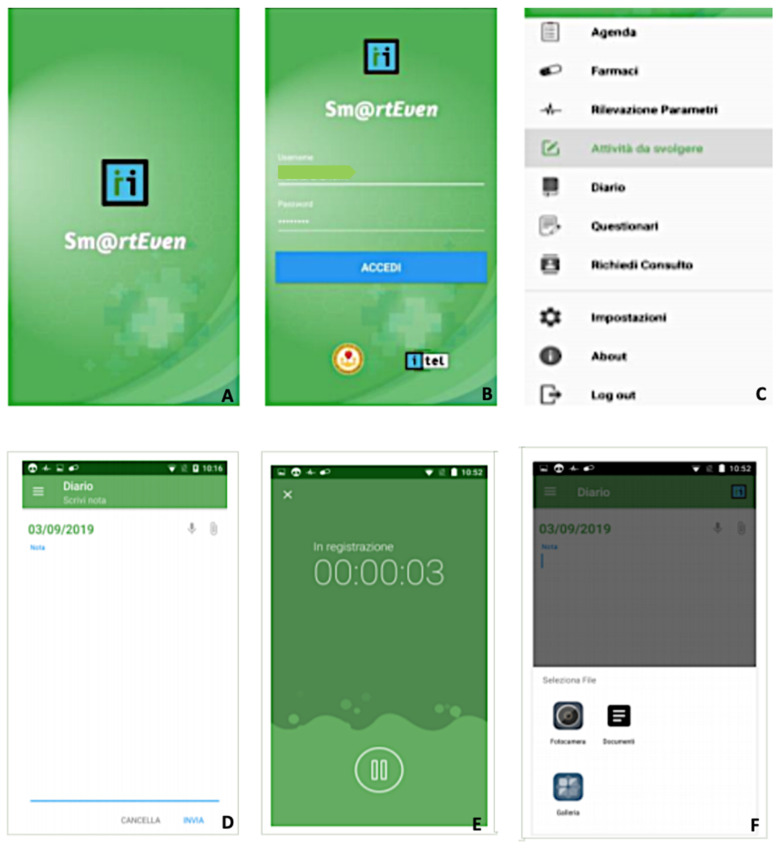
Sm@rtEven application: (**A**) initial web page; (**B**) screen for entering the patient’s personal data; (**C**) main menu of the application, with the list of available functions; (**D**–**F**) secondary screens showing the possibility of inserting a note and attaching audio files, documents or images. The application also makes it possible to record a short voice message or attach a file from the smartphone’s memory.

Pairing the wireless medical device with a smartphone requires Bluetooth activation. After the first access, the patient profile and the list of functions available for detecting the various parameters can be viewed. The main menu has the following functions:

- Diary: List of the activities that the patient must carry out on the current date (parameter detection, drug administration, etc.) and the activities still to be performed from the previous day.
- Drugs: List of drugs prescribed and drugs not taken in the previous days. In the diary, it is possible to note the assumption, postponement, or refusal of the prescribed drug therapy.
- Parameter detection: List of vital signs to be detected and the measurements left unfinished from the previous day. After synchronization between the application and the wireless device, the data transmission occurs automatically after confirmation by the patient. For the body temperature parameter, the patient must manually enter the value in the dedicated section through guided steps.
- Activities to be carried out: List of activities to be carried out (specialist visits, etc.) and activities that remained unfinished from the previous day.
- Journal: Allows the patient to enter a note and attach audio files, documents, or images. The application also allows the patient to record a short voice message or attach a file from the smartphone’s memory. Instant photos can be taken as well (Figure 3D–F).
- Questionnaires: In this section, the patient can fill in the scheduled daily questionnaires. The notification to remind the patient to fill in the questionnaire appears in the diary. There are three different types of answers: binary, multiple-choice, and open answers.

### 2.2. First Protocol

Once the patients were identified according to the inclusion criteria, they were enrolled, and the wireless devices were delivered.

The physician then created a file with the patient’s personal and clinical information on the application control system. Such a procedure generated the credentials for the patient to access the system. The activation of the parameter detection program occurred 24 h after the creation of the medical record.

The patient was asked to enter the required parameters every day. The number of steps per day was automatically recorded by the device. Another parameter to be evaluated daily was the measurement of the progressive load on the operated limb, expressed in kg, by means of the wireless scale. Additionally, in this case, the recording of the value was automatic and immediate due to the synchronization of the device with the patient’s smartphone. Body temperature measurement was taken twice a day, in the morning and evening, to record any fluctuations throughout the day.

In the case of the non-functionality of automatic transmission, the patient could enter the value manually, following a guided procedure.

The daily questionnaire is a compilation of ten questions:

1. Is the foot of the operated limb swollen?
2. Do you feel tingling in the operated limb?
3. Is the dressing in order?
4. Are there any secretions from the wound?
5. How many crutches are you using?
6. How are you feeling today?
7. Are you able to be autonomous in daily activities?
8. Do you have a fever?
9. Did you experience headaches or dizziness?
10. Describe all side effects.

The detection of the parameters was required for a period of 30 consecutive days (Table 1). To monitor the actual participation of patients in the project, staff members sent instant notifications in case of non-participation.

### 2.3. Second Protocol

A simplified protocol for the remote assistance project was proposed in consideration of the SARS-CoV-2 virus pandemic and the logistical problems encountered with the first protocol.

This revision was intended to enroll patients after more than 30 days of their operation. In this way, any protection from plaster casts or orthopedic orthoses (recommended in the first postoperative days) was removed and surgical wound dressings were no longer necessary.

However, it was decided to evaluate the same parameters as the first protocol. 

The frequency of measurements in the second protocol was reduced. The number of steps performed was still recorded daily. The measurements of the load and body temperature trend were reduced to twice a week (Table 2).

The second protocol saw a reduction in the number of questions featured in the daily questionnaire. The questions proposed were:

1. Is the foot of the operated limb swollen?
2. Do you feel tingling in the operated limb?
3. How many crutches are you using?
4. How are you feeling today?
5. Are you able to be autonomous in daily activities?
6. Do you have a fever?
7. Describe any side effects.

To obtain greater patient collaboration, the monitoring was reduced to 14 days compared to the expected 30 days. 

To simplify patient training in using the application, the patient was asked to install the application on their smartphone before making an appointment for the delivery of the wireless devices. During that appointment, the recruiter doctor showed the various functions of the application and clarified any doubts to the patient.

A simplified brochure was drawn up and sent by email to patients, along with the consent to participate in the study and the authorization to process sensitive personal data, before the devices were delivered. In this way, the patient could view the documentation provided within an appropriate time and then dispel any doubts during the appointment with the recruiter doctor.

In order to evaluate the effectiveness of the application herein analyzed, we assessed patient compliance (C) over time. Compliance lies in the degree of cooperativeness in the execution of the doctor’s prescriptions and emphasizes the satisfaction and collaborative spirit towards the therapeutic, rehabilitation, or pharmacological program set. The quality of communication between doctor and patient is the main determinant of patient compliance; it hinges on the doctor’s ability to understand the needs and circumstances experienced by patients, communicate with them in a straightforward but professional way, and provide technical clarifications and reassurances regarding the patient’s difficulties [11,12].

## 3. Results

Initially, a group of nine patients was recruited for a duration of 30 days: four women (45%) and five men (55%) who went through the first protocol. However, during the collection of the various parameters, some critical issues were highlighted, which led to the development of a new simplified protocol.

Among the various critical issues that emerged with the use of the first protocol was the need to have a smartphone with an Android operating system. This condition excluded some otherwise eligible patients, particularly young people with higher technology skills. In fact, out of 40 eligible patients, only 9 (22.5%) had a smartphone with an Android system. The number of patients that could be enrolled was also conditioned by a limited number of devices available: five activity trackers and five electronic scales. Enrolled patients had a mean age of 52 years (range 49–70). Difficulties in managing the application functions were found predominantly in four patients over 60 (n° 4). In addition, three patients (30%) experienced problems pairing the wireless devices with their smartphones.

Furthermore, the activation of the application 24 h after installation made it difficult to explain to the patient the correct functioning of the app during their time at the hospital as the average hospital stay was 24–36 h after surgery.

The program structure requires an average time of about 30 min to create the computerized medical record of each patient. The creation of a file that contains the patient’s clinical and anamnestic information is the exclusive task of the recruiting physician and is sometimes not very compatible with hospital medical activity, especially in consideration of the pandemic period.

After an initial phase of correct use of the application and devices, a reduction in patient compliance occurred over time.

− From the 1st to the 7th day: 100% of the required parameters and the appropriate filling-in of the questionnaire were recorded;
− From the 8th to the 14th day: registration of the number of steps was equal to 100% of the requested assessments, the temperature parameter to 60%, registration of the load granted on the operated limb to 60%, and completion of the questionnaire to 90%;
− From the 15th to the 21st day: registration of the number of steps was equal to 70% of the requested assessments, registration of the temperature parameter to 40%, registration of the load granted on the operated limb to 40%, and filling in the questionnaire to 60%;
− From the 22nd to the 30th day: the recording of the number of steps was equal to 30% of the requested assessments, recording of the temperature parameter to 10%, recording of the load granted on the operated limb to 10%, and filling in the questionnaire to 30%.

**Figure 4:**
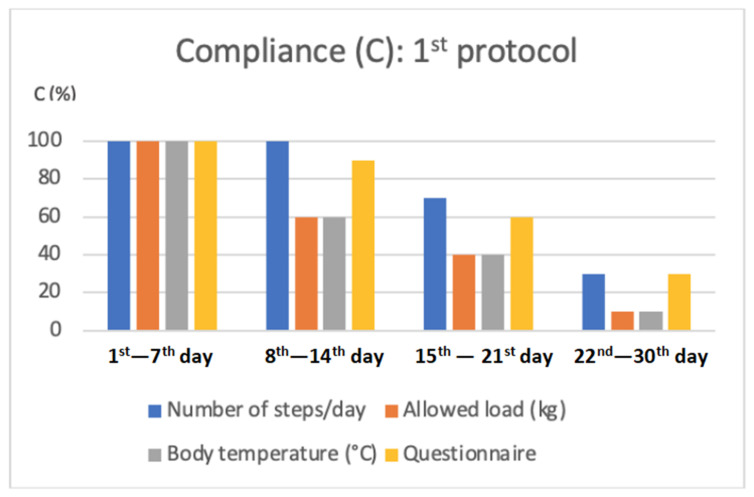
Graphical representation of the average compliance of patients, expressed as a percentage, in the insertion of the various parameters required within the first protocol.

Patient compliance in the first week was 100%, and in the second, it was 77.5%; in the third week, it was 52.5%, and in the fourth week, it was 20% (Figure 4).

In the next phase, with the second protocol, a group of 10 patients was recruited, 6 women (60%) and 4 men (40%), to whom the second protocol was applied for a total period of 14 days. The enrolled patients had a mean age of 55 years (range 49–70).

− From the 1st to the 7th day: 100% of the required parameters and the appropriate filling-in of the questionnaire were recorded;
− From the 8th to the 14th day: registration of the number of steps was equal to 100% of the assessments requested, registration of the temperature parameter to 100%, registration of the load granted on the operated limb to 90%, and completion of the questionnaire to 95%.

**Figure 5:**
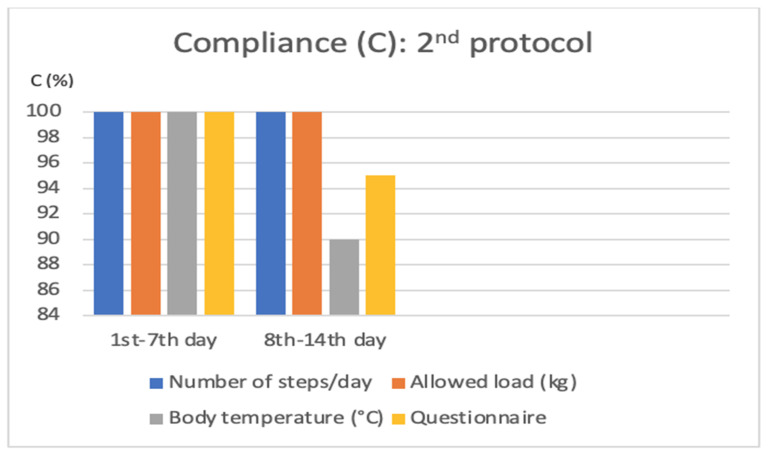
Graphical representation of average patient compliance, expressed as a percentage, in the insertion of the various parameters required within the second protocol.

Patient compliance in the second week was 96.25% in the recording of the required parameters (Figure 5).

## 4. Discussion

The innovation of technology has changed the way patients interact with medical services, offering individualized and participatory health services. Healthcare apps have been proven to be useful tools for evaluating the physical, functional, and psychological states of patients. Such applications can provide information to patients and to their caregivers and can help to monitor the patient’s adherence to pharmacological and rehabilitation programs [3,4,5,6,7,8,9]. 

These new technologies allow patients who have physical impediments that limit their access to health services to receive care remotely through telemedicine applications. Healthcare apps offer opportunities for access to care even to patients who reside in extremely populated areas, such as the metropolitan area of Milan, where the study was conducted (number of inhabitants 3,265,327/ISTAT- National Institute of Statistics Database, updated to 01.01.2020), disadvantaged areas, or areas with impediments that limit access to treatments [13].

Telemedicine is defined by the World Health Organization as “the provision of health services, in which distance is a critical factor, by all health professionals who use information and communication technologies for the exchange of valid information for the diagnosis, treatment, and prevention of diseases and accidents, research, and evaluation and for the continuous training of health professionals, all in the interest of promoting the health of individuals and their communities” [14].

This study aimed to evaluate the functioning of the Sm@rtEven application for the remote monitoring of patients suffering from a fracture of the lower limb over time. Some critical issues emerged during the monitoring, which led to the formulation of a second protocol to meet the patients’ needs.

In the first protocol, there was the possibility of monitoring the condition of the surgical wound. However, most patients who underwent reduction and fixation surgery for lower limb fractures were protected with a cast in the immediate postoperative period. This protection required a more complex dressing that could not be done by the patients themself. Although the monitoring of the surgical wound may be useful, the difficulties related to the anatomical site and the need to correctly position the plaster led to the suspension of this parameter in the second protocol as it could cause the onset of complications such as infections or pressure wounds. For this reason, in the second protocol, it was decided to suspend the remote evaluation of the surgical wound and maintain direct clinical checks until the complete healing of the wound and then focus our attention on the rehabilitation program. The presence of a cast also negatively affected the number of steps that could be performed, and this resulted in the absence of load in the first postoperative phase. For this reason, in the second phase, it was decided that only patients beyond 30 days after surgery would be enrolled.

The casts were removed, and the loads on the affected limb were applied after performing an X-ray check. We, therefore, prefer monitoring the rehabilitation phase rather than the first postoperative phase.

The results show a significant reduction in patient compliance over time with the first protocol. Sometimes, patients forgot to recharge and/or wear the activity tracker, with a partial loss of steps taken, or failed to adhere to the time slots indicated for entering the required parameters, or did not perform the required monitoring. The 30-day monitoring period required reduced the availability of patients. A decisive factor was the high number of required measurements associated with questionnaires, defined by patients as long and redundant. For these reasons, the number of measurements required and the length of the questionnaire in the second protocol were reduced.

The formulation and application of the second protocol attempted to respond to the critical issues that emerged during the first phase. First, the shorter duration of monitoring and the limited number of evaluations made patients more willing to participate in the project.

The second protocol was more functional and effective than the first because it made it possible to maintain greater patient compliance in compiling the required parameters in the second week compared to the first protocol (96.25% vs. 77.5%) and in the overall duration of the project (98.1% vs. 62.5%), allowing us to obtain a more truthful picture of the patient’s clinical condition. 

The reduction of the duration of the protocol (14 vs. 30 days) and the reduction of the total number of measurements required (36 vs. 150) ensured greater adherence to the project.

To overcome the problem of pairing wireless devices with their smartphones, patients can use other free and easily installed applications on their mobile devices to monitor the number of steps taken.

This also avoids the loss of values related to the need to recharge the activity tracker device. In criticism of the use of different devices, as they provide non-standard values, it should be emphasized that the instrument for measuring body temperature was also not standardized; in fact, this parameter was evaluated with different and personal tools and then the value was entered manually.

The modified protocol made it possible to follow patients in an important phase of the restitutio ad integrum process of daily functions and activities. This project is particularly valuable in the rehabilitation phase. The choice of recruiting patients in this phase made it possible to respond to the need to follow the patient during the various phases of functional recovery, where medical visits are reduced in frequency and the patient often feels abandoned. In fact, it allows the patient to stay in contact with medical staff during the convalescence phase (after passing the critical healing phase), which generally corresponds to the first postoperative month. Compared to traditional medicine, telemedicine offers its advantages mainly to populations living in remote regions or rural areas, where few doctors are available, enabling patients to access healthcare services by reducing the need to travel and the risks thereof [1]. However, such innovations also raise ethical challenges. The exchange of health information and the provision of telematic care could create new risks to the quality, safety, and continuity of care [1,15,16]. According to the provisions in Art. 78 of the 2014 Code of Medical Ethics [17].

*“... Computer-based digital technology... The doctor, in the use of IT tools, guarantees the acquisition of consent, the protection of confidentiality, the relevance of the data collected, and, to the extent of his competence, the safety of techniques. The doctor, in the use of information and communication technologies of clinical data, pursues clinical appropriateness and adopts his own decisions in compliance with any multidisciplinary contributions, ensuring the conscious participation of the assisted person. The use of information and communication technologies for the purposes of prevention, diagnosis, treatment, or clinical surveillance, or such as to affect human performance, adheres to the criteria of proportionality, appropriateness, efficacy, and safety, in compliance with the rights of the person and of the application addresses attached...”*

[18].

The Sm@artEven application meets all the above ethical requirements, in full guarantee of the confidentiality of personal data, the acquisition of valid consent, and, finally, the suitability of staff to use the digital tool [19,20]. As for the specific acquisition of consent, Italian Law 219/2017 [21] mandates that it be acquired “*in writing or through video recordings, or, for the person with disabilities, through devices that allow communication*”, enhancing the doctor–patient relationship and equally sharing time between communication and treatment; the Sm@rtEven app was designed to meet these legal requirements [1,22]. Lastly, as far as professional liability is concerned, it is worth bearing in mind what was remarked on 13 April 2020 by the Italian High Institute of Health in the document “*Interim indications for telemedicine assistance services during the COVID-19 health emergency*”, which also contains references regarding health responsibility:

*“… acting in telemedicine means assuming full professional responsibility, even for the smallest action carried out at a distance. Specifically, the correct management of limitations due to physical distance is part of the aforementioned responsibility in order to guarantee the safety and effectiveness of medical and assistance procedures, as well as compliance with the rules on data processing... In this context, also for the purposes of the management of clinical risk and health responsibility, the correct professional attitude consists in choosing the operational solutions that offer the best guarantees of proportionality, appropriateness, efficacy, safety, and respect of the rights of the person. In summary, it is not a question of choosing the technologies, but the doctor must choose the combination of them that appears the most appropriate from the medical-assistance point of view in the individual case... the execution of telemedicine... is unsafe when using digital tools in the patient’s home to carry out the video call. We remind you that it is clear that all the legislative and ethical rules of the health professions apply exactly to telemedicine health activities... even in not perfect practical conditions, it seems acceptable that the video call can be used by the doctor to support the clinical control of those patients he already knows from having previously visited them at least once…”*

[23].

Therefore, according to these indications, the doctor assumes responsibility for the health behaviors related to the services provided at a distance, also noting the possible insecurity of the devices used at home and highlighting that all the legislative and deontological rules of the health profession extend to telemedicine [1,19,20,24,25].

In this regard, Art. 7 of Law 24/2017 (the so-called “Gelli-Bianco” law) [26] establishes that health facilities are also responsible for the harmful consequences of health services provided in an autonomous intramural regime in the context of clinical research and experimentation, “as well as through telemedicine”.

Therefore, should unfair damage materialize due to telemedicine-delivered health services, such damage would be among those for which the health facility is required to answer; professionals would be held liable only in the event of wilful misconduct or gross negligence.

In the area of telemedicine, the potential professional responsibility profiles reside in:

− Production defects of the equipment;
− Errors in the installation or implementation of the various components of IT support;
− Omitted/defective/ineffective maintenance;
− Errors in the use of the equipment;
− Errors in data transmission.

### 4.1. European Norms Reflect Telemedicine Complexities

It is worth pointing out that the complexities and unique, distinctive traits of telemedicine have been addressed by numerous European Union regulatory initiatives over the years. As a healthcare service, telemedicine is deemed to fall within the Treaty on the Functioning of the European Union (TFEU), namely, Articles 56 and 57, which characterize the notion of a “service” being entitled to free movement (“restrictions on freedom to provide services within the Union shall be prohibited in respect of nationals of Member States who are established in a Member State other than that of the person for whom the services are intended”) as a general principle and not being “governed by the provisions relating to freedom of movement for goods, capital, and persons”.

However, since telemedicine is an information service (provided for remuneration, as in Art. 57 TFEU), but, at the same time, a healthcare service, both the set of norms applicable to healthcare and that governing Information Society services have a bearing under European statutes [27,28].

Directive 2011/24/EU, also known as the Cross-Border Directive, is the most relevant EU regulatory intervention governing health services, while information and telecommunications are broadly regulated by several directives, among which are the Directive on Services of the Information Society [29,30], the Electronic Commerce Directive [31,32], and the Privacy and Electronic Communications or e-Privacy Directive [33]. Liability for defective products is regulated at the EU level by Directive 85/374/EEC54, applicable to any piece of equipment either manufactured in or imported into the European common market. The fundamental goal of the said Directive is to guarantee a high level of consumer protection against possible health or property damages arising from any defective product. Pursuant to Article 6 of the Directive, a product is deemed defective when it does not ensure a degree of safety that would be reasonable to expect. Moreover, the Directive codifies the “objective/faultless liability” principle of the manufacturer or the importer. If no producer or importer can be identified, the supplier is to be held liable instead [34,35].

Significantly, the EU has taken several steps over the years to improve the access and availability of telemedicine among member countries and to enhance coverage of national healthcare systems by earmarking substantial research funding for the development of new optimized tools. A recent report by the commissioned advisory body eHealth Stakeholder Group, titled ‘Widespread Deployment of Telemedicine Services in Europe’, has elaborated on and highlighted the benefits of telemedicine [36]. The degree to which such innovations can be marketed has been explored and reported on by EIT Health, a network of best-in-class health innovators backed by the EU and the community of world-leading healthcare innovators in Europe. The report concluded that although the benefits of telemedicine for insular and remote regions are substantial, its broader, mainstream use throughout Europe is still far from possible. That is mostly due to the still high costs of setting up and performing telemedicine services. In addition, other issues arise from the inadequacy of technical infrastructure in the Member States, which causes interoperability impediments and confidentiality and privacy-related concerns over health data management. A still inadequate framework of ethical rules and proof of concept that is applicable to telemedicine, the sense of uncertainty of health professionals concerning possible exposure to liability and litigation, and, last but not least, the still hazy regulatory and legal frameworks are also issues to be addressed [37].

### 4.2. Considerable Advantages with a Few Caveats

The remote evaluation of patients is associated with indisputable benefits, such as a reduction in SARS-CoV2 infection, a decrease in healthcare costs, the facilitation of social distancing, and shorter waiting times. There is also a cost-saving for society as patients can use the app in their workplace or at home, minimizing the loss of productivity and improving the economy of movement. The need for travel is reduced, the cost of which is borne by the patient, the hospital, or the government. Furthermore, an ecological benefit can be expected since a reduction in travel can lead to an improvement of environmental conditions through the reduction of emissions [38]. However, some negative aspects persist, such as the anxiety of the doctor and the patient, mostly due to possible misunderstandings; the technology is not always adequate, and there are the medical–legal data vulnerabilities and privacy and security issues [39,40,41], i.e., many of the same issues posed by contact-tracing applications during the pandemic [42]. Experimental studies have highlighted the appreciation for video and telephone consultations, mostly thanks to the greater speed compared to face-to-face visits, but have also unveiled doubts about a lower quality of services, lower levels of understanding by patients, and concerns about the acceptability and benefits of using telemedicine with hearing-, visual-, or cognitive-impaired patients and with patients with limited health literacy [39,43]. Research findings also seem to be concordant that in defining the most suitable approach to each individual patient, the use of the telephone alone is not adequate, and the contact must always be audio-visual [35]. Using personal mobile devices through a Bring Your Own Device (BYOD) policy can potentially save significant costs to the healthcare system, whereby there is no need to purchase facility-owned devices. Patients can bring their own devices, eliminating the need for an additional or training device. BYOD policy is perceived as a key factor in balancing users’ convenience needs with institutional security. However, socio-technical interventions are necessary to create an environment for safe use of mobile devices through the development of BYOD policies that balance the need for user comfort with the safety of the organization and the privacy of patients. BYOD has become a leading approach in healthcare settings. With greater familiarity and reduced costs, BYOD can facilitate doctor–patient communication and interoperability. Moreover, mobile technology facilitates doctor–patient communication, data collection, and statistical analysis.

Real data, smooth doctor-to-patient communication, and online support can only improve patient outcomes and quality of life. In the end, patients are the core of research and digital health [44,45,46].

The characteristics of the Sm@rtEven application minimize the critical issues related to the type, operation, and management of technological equipment as well as those relating to the behavior of healthcare personnel.

## 5. Conclusions

The use of telemedicine has the same value as traditional clinical evaluations but enables patients to be followed in both the postoperative period and the rehabilitation phase [47,48,49,50]. It also facilitates the continuation of clinical monitoring over long distances without creating discomfort for patients with lower limb fractures.

The Sm@rtEven application has proved to be a valid tool for continuing to follow patients at a distance, especially in a pandemic, even though there was a low number of patients in this study. The limited number of patients was determined by several factors. The use of this application exclusively on the Android operating system reduced the number of young patients that could be recruited. Patients with degenerative cognitive diseases could not use the application. Other middle-aged patients refused to participate in the study due to a lack of confidence in using smartphone technologies at the time of recruitment. Moreover, during the pandemic, the limiting factors for recruitment included the number of admissions to the hospital for medical consultations being limited to severe cases, the average hospitalization time of patients being reduced (often, it was not enough for adequate training in the use of the application), and the limited number of wireless devices available and their maintenance time per patient.

Different benefits are associated with the use of this healthcare application, such as a reduction in SARS-CoV2 infection, lower healthcare costs, the ability to keep social distancing, shorter waiting times, and the environmental benefit due to the diminished need for traveling [51].

To make the system usable by a greater number of patients, it would be advisable not to use wireless devices but personal tools and applications that can be installed on the patients’ smartphones to monitor the parameters considered. Further changes will be needed to simplify the application and to allow the recruitment of older patients with less confidence in new technologies.

## Figures and Tables

**Table 1:** Number of measurements requested from each patient in the first protocol.

	Number of Steps/Day	Allowed Load (kg)	Body Temperature (°C)	Questionnaire	Total
Daily	1	1	2	1	5
Weekly	7	7	14	7	35
Monthly	30	30	60	30	150

**Table 2:** Number of measurements requested from each patient in the second protocol.

	Number of Steps/Day	Allowed Load (kg)	Body Temperature (°C)	Questionnaire	Total
1st week	7	2	2	7	18
2nd week	14	4	4	14	36

## Data Availability

The data presented in this study are available upon request from the corresponding author.
